# Prevalence of TLR4 single nucleotide polymorphisms (ASP299GLY, THR399ILE) in healthy subjects and septic patients, and association with outcome

**DOI:** 10.1186/cc10610

**Published:** 2012-03-20

**Authors:** T Mohovic, R Salomao, E Nogueira

**Affiliations:** 1 Albert Einstein Hospital, São Paulo, Brazil; 2 UNIFESP, São Paulo, Brazil

## Introduction

Our study aimed to determine the prevalence of functional SNPs (Asp299Gly, Thr399Ile) of TLR4 receptors, in healthy volunteers and septic patients in a Brazilian population and to correlate the presence of these polymorphisms in septic patients with clinical outcome.

## Methods

We verified the presence of polymorphisms ASP299GLY, THR399 ILE by PCR-restriction fragment length polymorphism followed by digestion with enzymes *Nco*I for SNP 299 and *Hinf*I for SNP399 followed by electrophoresis for identification of alleles.

## Results

We observed a statistically significant difference between the genotypes of the Thr399Ile polymorphism and respiratory dysfunction, indicating a higher frequency than wild-type genotype in subjects with respiratory dysfunction than those without this condition (*P *= 0.001). We also observed a statistically significant difference between genotype groups formed by the Asp299Gly and Thr399Ile polymorphisms and respiratory dysfunction more often featuring group 299Selv/399Selv grupo299Het/399Het and less frequently in individuals with respiratory dysfunction than those without this condition (*P *= 0.003).

## Conclusion

Our study shows for the first time an assessment of the prevalence of polymorphisms of TLR4 Asp299Gly and Thr399Ile considering its cosegregation in healthy individuals and septic patients. And that septic patients who develop respiratory dysfunction have more presence and genotypes 399Selv 299Selv/399Selv and less the presence of genotype 299Het/399Het, featuring a protective effect of the polymorphism Thr399Ile.
